# ICG Flow 800 technology targeted STA-MCA microvascular bypass for exclusion of deep-seated fusiform MCA aneurysm: 2-dimensional operative video

**DOI:** 10.3171/2021.10.FOCVID21183

**Published:** 2022-01-01

**Authors:** Carlos Candanedo, Kobi Goldstein, José E. Cohen, Sergey Spektor

**Affiliations:** 1 Department of Neurosurgery, Hadassah-Hebrew University Medical Center, Jerusalem; and; 2 Surgical Monitoring Services Ltd., Beit Shemesh, Israel

**Keywords:** dissecting aneurysm, EC-IC bypass, ICG FLOW 800, middle cerebral artery, video

## Abstract

The authors present the case of an 18-year-old male with a deep-seated left fusiform dissecting M3 aneurysm for which endovascular treatment was not applicable. At the open surgery, they used the less commonly reported FLOW 800 fluorescent indocyanine green (ICG) videoangiography, before and after parental aneurysmal artery temporary clipping, to locate the distal outflow branch of the aneurysm and use it as the recipient artery for a superficial temporal artery–M4 bypass, excluding the aneurysm by clipping the parental artery. Repeated ICG FLOW 800 angiography confirmed bypass patency and adequate blood flow. The aneurysm’s exclusion from circulation was confirmed by digital subtraction angiography postoperatively.

The video can be found here: https://stream.cadmore.media/r10.3171/2021.10.FOCVID21183

## Transcript

This is Dr. Carlos Candanedo from Hadassah-Hebrew University Medical Center in Jerusalem, Israel. This video demonstrates a noncommonly use of indocyanine green (ICG) FLOW 800 fluorescent angiography technology to correctly identify the distal vessel of a fusiform dissecting aneurysm to be bypassed when this aneurysm needs to be excluded from circulation.^1–7^

### 0:43 Patient Clinical History and Neurological Exam.

We present a case of an 18-year-old male who started to suffer from repetitive transient right facial and arm numbness and weakness events.

### 0:53 Imaging Studies.

MRI showed a left M3 fusiform dissecting aneurysm with diffuse small white matter infarcts in the frontoparietal lobes.

### 1:02 Concept of Treatment.

Due to the distal location of the aneurysm and the small diameter of the arteries, endovascular treatment was not an option. Instead, we planned the open surgery to perform microvascular bypass of the superficial temporal artery with the distal outflow branch of the aneurysm to confirm the anastomosis patency and, after that, to ligate the parental branch of the aneurysm, excluding it from the flow. We assumed that ICG FLOW 800 fluorescent angiography would help us identify the correct cortical artery that will be used for the anastomosis.

### 1:39 Patient Positioning.

The surgery was performed with the patient supine, with the head turned to the right side. We used neuromonitoring, including MEP, SSEP, and EEG. With the Doppler ultrasound, we recognized the frontal and parietal branches of the superficial temporal artery. We made a curvilinear skin incision starting in the left frontal area, curving frontotemporally and ending behind the pinna, suitable for harvesting either frontal or ascending parietal branch of the STA and convenient at the same time for a frontotemporal craniotomy.

### 2:16 Donor Artery Preparation and Craniotomy.

After galeal flap elevation, we harvested the ascending parietal branch of the superficial temporal artery, leaving it open in the flap without ligation. Then frontotemporal craniotomy was made.

### 2:29 Dura Opening and Sylvian Fissure Dissection.

After dural opening, we proceeded with arachnoid dissection. In this case, we must open the sylvian fissure wide to provide the optimal conditions for the aneurysm exposure and identify and understand the vascular anatomy. Identified the left optic nerve and laterally the left internal carotid artery. Now the first segment of the middle cerebral artery, M1. This is the MCA bifurcation, with an early temporal branch, the main temporal, and frontal branches. Finally, the entire sylvian fissure was exposed wide, exposing the MCA and its bifurcation.

### 3:15 M3 Fusiform Dissecting Aneurysm Exposure.

As we follow the M2 branches, we approached the aneurysm that we can see now, big size, without neck, firm, and calcified. We started to dissect carefully around the dome of the aneurysm. The vein of Labbé limited the distal exposure of the sylvian fissure very much, and we had to transect a minor tributary vein to improve the exposure. Now we see much better most of the aneurysm, but we cannot identify outflow vessel yet, because the exposure is limited by the vein of Labbé, which we cannot sacrifice.

### 3:54 ICG FLOW 800 Injection.

Now we see the parental branch of the aneurysm, and injected the indocyanine green, and identified two distal vessels, but now we do not know which is coming from the aneurysm.

We placed a temporary clip to the parental artery proximal to the aneurysm. Now we identified there is no filling of this distal branch, meaning that it is the continuation of the outflow branch of the aneurysm. Now we know which vessel should be used as a recipient artery for the anastomosis with the STA.

### 4:32 Recipient Artery Preparation.

We prepared the cortical recipient vessel with an approximately 1-mm diameter and dissected it from the pial vessels. We placed a small sheet of rubber glove under the vessel. Then, approached the donor vessel, ligated its distal branches, and dissected it entirely from the flap. Placed a proximal clip on the donor vessel and flushed it with heparin solution. We decided to select the anterior branch of the parietal STA as the donor vessel since its length was better to reach the bypass and ligated the other branch.

### 5:11 Microvascular EC-IC Bypass

 We made a fishmouth cut of the donor vessel. Placed a small silastic stent and pass the first stitch with Ethilon 10-0, which will be in the heel corner of the future anastomosis. We made a blue line over the side of the recipient vessel to easily identify the edges of the vessel’s wall. Only now we asked for burst suppression to decrease occlusion time during anastomosis and clipped the segments of the recipient vessel. After this, we made a longitudinal incision of the recipient artery. It is crucial that the length of the incision will match ideally to the length of the opening of the donor vessel. We placed another stent inside the cortical vessel, making the suturing easier. We accomplished the suture in the heel corner, which will be the first suture of the anastomosis. We finished this first stitch on the heel and added two other stitches at both sides to reinforce this heel corner. Then we performed a stitch in the toe corner and added two stitches on both sides while paying maximal attention for appropriate approximation of the vessel walls, intima to intima. We retrieve the stent and finish the bypass with additional stitches. An end-to-side anastomosis was done, 13 stitches were placed, with an occlusion time of 30 minutes. We removed both clips from the recipient artery and then unclipped the donor vessel.

There is slight bleeding, which is stopped with little pressure on the tiny bleeder.

### 6:49 Aneurysm Exclusion.

Then we approach the aneurysm again. Here we are able to identify the parent vessel of the aneurysm—the M3 branch of the MCA. At this step, the aneurysm ruptured, and bleeding started. We placed a clip on the aneurysm base and its parent vessel, but the bleeding continued due to the backflow. We placed a temporary clip on the distal M2 parent vessel, which reduced the bleeding but did not stop it completely. After that, we placed a large, strong clip across the body of the aneurysm, which stopped the bleeding. The temporary clip was uneventfully removed. We verified the preservation of the blood flow inside the M2 and contiguous M3 segment with Doppler US after temporary clip removal. No flow was revealed inside the aneurysm with the Doppler probe.

### 7:39 Postbypass ICG FLOW 800 Injection.

Finally, ICG was again injected, evidencing excellent patency of the bypass from the donor vessel. The aneurysm was not filled. The FLOW 800 delay map showed an excellent flow velocity and filling of the bypass and the brain parenchyma. We tried to place a clip distal to the aneurysm, but the vein of Labbé limited our view and did not permit additional distal sylvian fissure dissection. Burst suppression was ceased, and SSEP and MEP recovered to the baseline.

### 8:07 Closure.

We covered the dural defect with a collagen matrix. Bone flap placed back after remodeling the inferior part to avoid compression of the donor artery pedicle and muscles.

### 8:17 Postoperative Course.

The patient woke up without any focal motor deficit. However, despite prophylactic medications, he developed partial seizures attacks, which were finally well controlled without consequences. Immediate postoperative angiogram showing exclusion of the aneurysm, with patency of the bypass, however, a minimal retrograde filling of the distal end of the aneurysm. Postoperative MRI without any acute ischemic area on DWI and late angiogram showing good patency of the parental M2 and distal filling with minimal retrograde filling of the distal end of the aneurysm, which could not be approached without the injury of the vein of Labbé. Repeated DSA in 1 year confirmed excellent bypass function with the diminishing of the aneurysmal tail filling. Red arrows show the avascular area in the eloquent frontal lobe seen in the selective ICA injection, as it receives supply from the external carotid artery injection, filling through the STA bypass.

The patient is asymptomatic without any neurological deficit. We considered performing endovascular obliteration of the residual aneurysmal former outflow vessel using the extra-intracranial anastomosis as a route for the microcatheter. However, after a thorough discussion with the patient and given positive angiographic dynamics, he preferred to be followed only with repeated CT angiographies.

## Disclosures

The authors report no conflict of interest concerning the materials or methods used in this study or the findings specified in this publication.

## Author Contributions

Primary surgeon: Spektor. Assistant surgeon: Candanedo. Editing and drafting the video and abstract: Spektor, Candanedo. Critically revising the work: Spektor, Candanedo, Cohen. Reviewed submitted version of the work: Spektor, Candanedo, Cohen. Approved the final version of the work on behalf of all authors: Spektor. Supervision: Candanedo. Neurophysiologist: Goldstein.
